# The Relation Between Precarious Employment Arrangements and Social Precarity: Findings from the PREMIS Study in Stockholm, Sweden

**DOI:** 10.1177/00207314211051880

**Published:** 2021-11-24

**Authors:** Nuria Matilla-Santander, Johanna Jonsson, Bertina Kreshpaj, Cecilia Orellana, Joan Benach, Kathryn Badarin, Bo Burström, Alejandra Vives, Katarina Kjellberg, Susanne Strömdahl, Gun Johansson, Per-Olof Östergren, Theo Bodin

**Affiliations:** 1 Unit of Occupational Medicine, Institute of Environmental Medicine, 27106 Karolinska Institutet, Sweden; 2 Health Inequalities Research Group, Employment Conditions Knowledge Network (GREDS-EMCONET), 16770 Universitat Pompeu Fabra, Barcelona, Spain; 3 Johns Hopkins University—Pompeu Fabra University Public Policy Center, Barcelona, Spain; 4 Transdisciplinary Research Group on Socioecological Transitions (GinTRANS2), Universidad Autónoma Madrid, Madrid, Spain; 5 Equity and Health Policy Research Group, 27106 Karolinska Institutet, Sweden; 6 School of Medicine, CEDEUS, 28033 Pontificia Universidad Católica de Chile, Santiago, Chile; 7 Centre for Occupational and Environmental Medicine, Region Stockholm, Stockholm, Sweden; 8 Section of Infectious Diseases, Uppsala University, Sweden; 9 27106 Karolinska Institutet, Sweden; 10 Social Medicine and Global Health, 5193 Lund University, Sweden

## Abstract

Precarious employment (PE) is a well-known social determinant of health and health inequalities. However, as most previous studies have focused on physical and mental well-being, less is known about the social-related outcomes (ie, social precarity) associated with precarious arrangements. This cross-sectional study aims to investigate whether PE is associated with social precarity in a working population of 401 nonstandard employed workers in Stockholm, Sweden (2016-2017). PE was assessed with the Swedish version of the Employment Precarious Scale (EPRES-Se) and analyzed in relation to social precarity related to working life (eg, task quality and job security) and living conditions (eg, restraint in social activities and financial constraints). We found positive adjusted associations between quartiles of EPRES-Se and social precarity related to working life (eg, being locked in an occupation [aPR_q4_:1.33 [1.10-1.61]]) and living conditions (eg, inability to participate in social activities because of work [aPR_q4_:1.27 [1.10-1.46]]). Our findings suggest that individuals in PE experience social precarity, stressing that PE may have negative effects on well-being. Further studies using multidimensional constructs of PE and larger samples should analyze these findings according to social and policy contexts in order to be able to inform policymakers.

## Introduction

### Precarious Employment

As a consequence of major social, economic, and political changes that took place in the mid-1970s, the overall quality of employment relationships has decreased over the past decades.^1^ This gradual transformation of the so-called standard employment relationship (i.e., full-time, secure employment with collective bargaining power) into nonstandard and atypical employment has bolstered the rise of precarious employment (PE).^2^ PE is commonly defined as an accumulation of unfavorable facets of employment quality, such as employment instability (eg, temporary employment, subcontracting, and multiple job holding), lack of power and rights (eg, asymmetric power relations and inability to exercise rights), and poor terms (eg, low wages and lack of benefits and/or training).^3,4^

Usually, the population groups most affected by PE are women, young workers, immigrants, manual workers, and workers with low educational attainment.^5^ PE has multiple consequences, which include a higher likelihood of being exposed to work environment hazards (eg, psychosocial, ergonomic, and physical hazards), suffering from material deprivation (ie, inadequate resources to maintain living standards, poverty, and life insecurity), and experiencing low-quality employment arrangements (eg, boring or dissatisfying work, psychological perceptions of job insecurity).^6^

### Employment Precarity and Social Precarity

PE is a well-known social determinant of health and health inequalities.^4–7^ However, as most previous studies have focused on physical and mental well-being, less is known about the social-related outcomes (ie, social precarity) associated with PE arrangements. Social precarity, a concept previously used by other authors, can be defined as the factors related to higher risks of social exclusion and has two dimensions: living conditions (ie, poverty, financial resources, social connections, social isolation, and satisfaction with family life) and working life (ie, task quality, work pressure, skill development, and job security).^8^

Various forms of nonstandard employment arrangements and single dimensions of PE have been associated with a wide range of social precarity measures, such as a reduced likelihood of having children in the future,^9,10^ poorer work–life balance, greater family conflicts,^11^ poor household income situation,^12^ and financial strain.^13^ It is important to note that studies exploring social consequences of PE measure PE through perceived job insecurity. However, job insecurity is actually a cognitive/affective phenomenon and a consequence of PE (experience of precariousness) rather than a characteristic of the quality of the employment relationship in itself.

### The Need for a Multidimensional Perspective

Although the aforementioned studies provide useful information, it is relevant to consider that nonstandard employment arrangement is not synonymous with PE. Thus, a consideration of multiple dimensions of the employment arrangement is needed to correctly classify employment arrangements as precarious. Multidimensional measures of PE may capture better all the aspects of the employment relationship and its quality.^14,15^ To our knowledge, only two Canadian studies have explored social precarity associated with PE using a multidimensional construct (the precarity index).^16^ These studies found that workers in PE are more likely to suffer social isolation, barriers for access to childcare (limiting the ability of both parents to work for pay), and poorer household well-being.

### Precarious Employment in a Nordic Welfare State Regime

The potential effects of PE are dependent (modifiable) on the policy (ie, institutional and regulatory protection, welfare regime) and social context (ie, axes of inequality, and resources and social support).^6^ Interestingly, a qualitative study that recruited workers using a multidimensional concept of PE from different contexts (England, Sweden, and Italy) found that some workers in PE do not experience social precarity (financial strain and poor task quality). The authors argued that this finding was related to the policy and social context of the workers.^17^ Nordic welfare regimes (Sweden) are characterized by reliable social protection measures, whereas other welfare regimes, such as liberal (England) or southern (Italy) regimes, are characterized by weak or highly fragmented social protection regulations.^18^ Therefore, we believe it is necessary to explore the association of PE and social precarity in Sweden (policy and social Nordic context) using a multidimensional construct.

### Aim

Against this background, this study aims to investigate whether PE (measured through a multidimensional construct) is associated with both dimensions of social precarity related to working life and living conditions in a working population of non-standard employed workers in Stockholm, Sweden.

## Methods

### Study Population and Data Collection

This cross-sectional study is based on data obtained from the PRecarious EMployment in Stockholm (PREMIS) study and the Longitudinal Integrated Database for Health Insurance and Labour Market Studies (LISA in the Swedish acronym) for the years 2016 to 2017. PREMIS is a study among nonpermanent workers (18-62 years) residing in Stockholm county between November 2016 and May 2017.^19^ The study recruited 483 individuals, but after exclusions, a total of 415 eligible individuals were included. The recruitment of participants and data collection was performed through web-based, respondent-driven sampling (non-probability sampling). An online survey included questions related to employment conditions (Swedish Employment Precariousness Scale [EPRES-Se]), work environment, health, life situation, and social networks. Details on PREMIS recruitment methods and study population can be found elsewhere.^19^ LISA is held by Statistics Sweden and integrates existing data from the labor market, sociodemographic characteristics, and so forth. Data collected with the PREMIS survey were linked to LISA by Statistics Sweden by means of the personal identification number unique to every person registered in Sweden and later de-identified.^20^

For the present study, we excluded individuals with missing values in any dimension of the EPRES-Se (n = 14).

### Study Variables

#### Exposure Variable: Precarious Employment

The level of PE was estimated using the Swedish EPRES-Se questionnaire,^21^ an adaption of the original Spanish EPRES-Es.^22^ The questionnaire included 23 items, grouped in six dimensions (temporariness, disempowerment, vulnerability, rights, the capacity to exercise rights, and wages). Each dimension was recoded to a 0 to 4 scale and thereafter its arithmetic mean was calculated. Next, we calculated the overall EPRES-Se mean score that could range from 0 (not precarious) to 4 (most precarious). The EPRES-Se score was used both as a continuous variable and divided into quartiles to estimate the level of PE. A separate overall EPRES-Se score was calculated to explore the financial constraint outcomes, excluding the “wages” dimension.

#### Outcome Variables: Social Precarity

The outcomes of this study are the two dimensions of social precarity related to working life and living conditions, measured through several variables.

Social precarity related to working life is measured through:

- Being locked in a job (current job situation does not suit them [no/yes]);
- Being locked in an occupation (prefer to work in something else [no/yes])^23^;
- Prefer to have permanent employment (no/yes);
- Difficulties in piecing several jobs together (response categories “no [never]” and “yes [always, often, and sometimes”]).

Social precarity related to living conditions is measured through:

- Restraint in social activities, including inability to participate in social activities because of work (no/yes) and avoiding talking about one's work situation in social contexts (no/yes);
- Financial constraints, including difficulties in managing regular expenses in the past 12 months, with the response categories “no” and “yes (once, more than once),” and inability to afford social activities, with the response categories “no (never)” and “yes (always, often, and sometimes)”

#### Covariates

The sociodemographic variables were: sex, age (18–24, 25–29, 30–35, and 35–62 years old), educational level (high school, higher education of ≤2 years, and higher education of ≥3 years), country of birth (Sweden, non-Sweden), family composition (cohabiting with children, cohabiting without children, single with children, single without children), and family disposable income (categorized in quartiles). Past employment conditions were explored with the number of jobs in the previous three years (1, 2–4, 5 or more) and having been unemployed in the past three years (no/yes).

We constructed a Directed Acyclic Graph (DAG) for drawing the assumed associations between PE, outcomes, and covariates (see Figure S1, Supplemental Material) using “DAGitty”.^24^ The minimal sufficient adjustment variables were sex, age, level of education, and country of birth.

### Statistical Analysis

We calculated the mean values of the EPRES-Se score and percentages of EPRES-Se score quartiles with 95% confidence intervals (95% CI) by sociodemographic characteristics. We later constructed box plots of EPRES-Se scores for each outcome.

Further, we calculated the prevalence of the outcomes with their 95% CI by the EPRES-Se score quartiles. We used generalized linear models, with the Poisson family and robust variances, for estimating the crude and adjusted prevalence ratios (PR, aPR) of presenting an outcome according to the continuous EPRES-Se score values and also, according to the EPRES-Se score quartiles, having the quartile with lowest EPRES-Se score (quartile 1) as reference. When the absolute frequencies were <10 in quartile 1, quartile 4 was used as the reference quartile.

Further, as the sample was recruited with respondent-driven sampling (RDS),^19^ weighted analyses were conducted in addition to the unweighted analyses. RDSII weights were calculated in RDS Analyst 0.42 for Windows. We conducted the analysis using Stata 16.0 statistical software.

### Ethical Considerations

The study was approved by the regional ethics board of Stockholm (dnr: 2016/1291-31/5). Written informed consent was attained by the respondent clicking “Yes” to the question “I understand the information given above and want to participate” after reading the study information. Personal identification numbers were replaced by serial numbers and stored separately after data collection.

Data was stored on password-protected, encrypted servers.

## Results

This study included 401 individuals: 54% were women, 43.9% were between 25 and 29 years, 38.9% had lower levels of education, and 80% were born in Sweden.

Table 1 shows the characteristics of the study participants by EPRES-Se score quartiles and EPRES-Se mean score. According to the sociodemographic characteristics, the most precarious employees—being those from the quartiles with the high EPRES-Se score (Q3 and Q4)—were men, young (18-24 years old), individuals with lower levels of education, born outside of Sweden, and had lower levels of family disposable income. Further, the most precarious employees had a history of multiple jobs and had been unemployed in the past three years.

**Table 1.** Characteristics of the study participants by EPRES-Se score quartile and EPRES-Se mean score.

			**Low** **precariousness (Q1: 0.09–1.54)**	**Medium-low precariousness (Q2: 1.55–1.90)**	**Medium-high precariousness (Q3: 1.91–2.25)**	**High precariousness (Q4: 2.26–3.07)**	**EPRES-Se mean**
		**n**	**% (95% CI)**	**% (95% CI)**	**% (95% CI)**	**% (95% CI)**	**Mean (95% CI)**
	**Sex**						
	***Men***	184	41.8 (32.4–51.9)	38.1 (28.9–48.2)	53.8 (44.2–63.1)	49 (39.3–58.8)	1.94 (1.87–2.02)
	***Women***	217	58.2 (48.1–67.6)	61.8 (51.7–71)	46.2 (36.9–55.8)	51 (41.2–60.7)	1.89 (1.82–1.96)
	**Age**						
	***18 to 24***	119	12.2 (7.05–20.4)	25.8 (17.9–35.4)	33 (24.7–42.6)	47 (37.4–56.8)	2.13 (2.05–2.21)
	***25 to 29***	176	50 (40.1–59.8)	45.4 (35.7–55.4)	42.4 (33.3–52.1)	38 (28.9–47.9)	1.87 (1.79–1.94)
	***30 to 35***	61	19.4 (12.7–28.5)	21.6 (14.5–31)	14.1 (8.7–22.2)	6 (2.7–12.8)	1.72 (1.61–1.83)
	***36 to 62***	45	18.4 (11.8–27.4)	7.2 (3.4–14.4)	10.4 (5.8–17.8)	9 (4.7–16.5)	1.80 (1.63–1.98)
	**Education level**						
	***High school***	156	31.9 (23.4–41.9)	37.9 (28.6–48.1)	41.7 (32.6–51.5)	49.5 (39.4–59.6)	1.98 (1.89–2.06)
	***Higher education* ≤ *2 years***	95	25.8 (17.9–35.4)	17.9 (11.4–27)	28.1 (20.3–37.7)	25.8 (17.9–35.7)	1.93 (1.83–2.03)
	***Higher education* ≥ *3 years***	137	42.3 (32.8–52.3)	44.2 (34.5–54.4)	30.1 (21.9–39.7)	24.7 (16.9–34.6)	1.80 (1.72–1.88)
	**Country of birth**						
	***Sweden***	321	85.6 (77–91.3)	84.5 (75.8–90.5)	84.8 (76.5–90.5)	68.4 (58.4–76.8)	1.87 (1.82–1.93)
	***Non-Sweden***	76	14.4 (8.7–22.9)	15.5 (9.5–24.2)	15.2 (9.5–23.5)	31.6 (23.1–41.5)	2.07 (1.95–2.20)
	**Family disposable income (yearly, 2016**–**2017, [SEK])**						
	***Q1 (7100***–***186 200)***	95	22.7 (15.4–32.1)	22.1 (14.8–31.7)	21.1 (14.3–30.1)	31.9 (23.2–42.1)	1.99 (1.89–2.10)
	***Q2 (186 900***–***257 300)***	100	22.6 (15.4–32.1)	28.4 (20.2–38.4)	25.9 (18.3–35.3)	25.5 (17.7–35.4)	1.94 (1.85–2.03)
	***Q3 (257 800***–***346 100)***	97	34.1 (25.2–44.1)	21.1 (13.9–30.5)	25.9 (18.4–35.3)	18.1 (11.5–27.2)	1.79 (1.68–1.89)
	***Q4 (347 400***–***2 296 000)***	98	20.6 (13.6–29.9)	28.4 (20.2–38.4)	26.9 (19.2–36.3)	24.5 (16.8–34.2)	1.90 (1.80–2.01)
	**Number of jobs in the past 3 years**						
	***1***	106	32.6 (24.1–42.6)	30.9 (22.5–40.9)	19.8 (13.2–28.6)	23 (15.7–32.3)	1.81 (1.73–1.90)
	***Between 2 and 4***	211	45.9 (36.2–55.9)	50.5 (40.6–60.4)	56.6 (46.9–65.8)	57 (47.1–66.4)	1.96 (1.89–2.02)
	***5 or more***	84	21.4 (14.3–30.7)	18.5 (11.9–27.6)	23.6 (16.4–32.6)	20 (13.2–29.1)	1.94 (1.82–2.06)
	**Unemployed in the past 3 years**						
	***No***	208	62.2 (52.2–71.3)	65.6 (55.5–74.5)	36.8 (28.1–46.4)	45 (35.5–54.9)	1.82 (1.75–1.89)
	***Yes***	192	37.7 (28.7–47.8)	34.4 (25.5–44.5)	63.2 (53.6–71.9)	55 (45.1–64.5)	2.02 (1.95–2.08)

Abbreviations: PR, prevalence ratio; aPR, adjusted prevalence ratio; CI, confidence interval.

**Figure 1.**
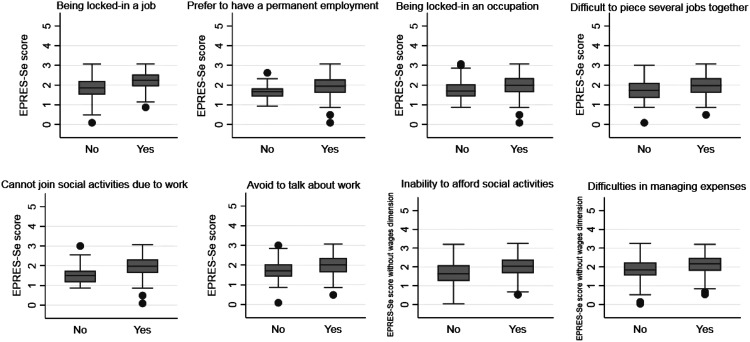
Median values of the EPRES-Se score according to social precarity related to working life and living conditions.

Figure 1 shows the median values of the EPRES-Se score according to social precarity related to working life and living conditions. The individuals with any of these outcomes were also those with the highest median values of the EPRES-Se score.

Table 2 shows the associations between social precarity and the EPRES-Se score quartiles. As EPRES-Se scores increase, so did the prevalence of declaring any experience of social precarity related to working life. This pattern was consistent in the crude and adjusted estimates; a high degree of PE was associated with preferring permanent employment (aPR_q4_:1.10 [1.01–1.19]) and being locked in an occupation (aPR_q4_:1.33 [1.10–1.61]). Regarding the restraints in social activities, we describe a steady pattern of higher prevalence of these as EPRES-Se scores increase. This pattern was consistent in the crude and adjusted estimates: inability to participate in social activities because of work (aPR_q4_:1.36 [1.19–1.56]) and avoiding talking about their working situation in social contexts (aPR_q4_:1.34 [1.09–1.65]). The associations of social precarity related to working life and restraint in social activities according to EPRES-Se score mean followed the same pattern (Table S1). As EPRES-Se scores increased, so did the prevalence of declaring financial constraints outcomes. These patterns were consistent in the crude and adjusted estimates: having difficulties in managing regular expenses (aPR_q4_: 2.26 [1.55, 3.30]) and not being able to afford social activities (aPR_q4_:1.27 [1.09, 1.48]). The associations of financial constraints outcomes according to EPRES-Se were also consistent with results using the score in a continuous manner (Table S1).

**Table 2.** Associations between social precarity and the EPRES-Se score quartiles.

				Precarious employment (EPRES-Se score quartiles*)			
			**Overall**	**Low precariousness (Q1: 0.09-1.54)**	**Medium-low precariousness (Q2: 1.55-1.90)**	**Medium-high precariousness (Q3: 1.91-2.25)**	**High precariousness (Q4: 2.26-3.07)**
**Social precarity related to working life**	**Being locked-in a job**	**n(%)**	56 (13.9%)	3 (3.1%)	10 (10.3%)	16 (15.1%)	27 (27%)
		**PR (CI95%)**	–	0.11 (0.03,0.36)	0.38 (0.19,0.75)	0.56 (0.32,0.97)	ref
		**aPR (CI95%)**	–	0.08 (0.03,0.27)	0.37 (0.18,0.72)	0.51 (0.29,0.90)	ref
	**Being locked-in an occupation**	**n (%)**	291 (73.1%)	58 (59.8%)	67 (69.8%)	82 (78.1%)	84 (84%)
		**PR (CI95%)**	–	ref	1.17 (0.95,1.44)	1.31 (1.08,1.58)	1.40 (1.17,1.69)
		**aPR (CI95%)**	–	ref	1.14 (0.92,1.41)	1.25 (1.03,1.52)	1.33 (1.10,1.61)
** **	**Would rather have permanent employment**	**n (%)**	370 (93.7%)	86 (90.5%)	85 (87.6%)	102 (98.1%)	97 (97.9%)
		**PR (CI95%)**	–	ref	0.97 (0.88,1.07)	1.08 (1.01,1.16)	1.08 (1.01,1.16)
		**aPR (CI95%)**	–	ref	0.97 (0.87,1.08)	1.09 (1.01,1.19)	1.10 (1.01,1.19)
** **	**Difficult to piece several jobs together**	**n (%)**	309 (77.2%)	67 (68.4%)	69 (71.1%)	84(80%)	89(89%)
		**PR (CI95%)**	–	ref	1.04 (0.86,1.25)	1.17 (0.99,1.38)	1.30 (1.12,1.51)
		**aPR (CI95%)**	–	ref	1.02 (0.85,1.23)	1.15 (0.97,1.36)	1.30 (1.12,1.51)
**Social precarity related to living conditions**	**Restraint in social activities**						
** **	**Cannot participate in social activities because of work**	**n (%)**	348 (87%)	69 (70.4%)	82 (84.5%)	100 (95.2%)	97 (97%)
		**PR (CI95%)**	–	ref	1.21 (1.04,1.40)	1.27 (1.10,1.46)	1.30 (1.13,1.49)
		**aPR (CI95%)**	–	ref	1.20 (1.03,1.39)	1.25 (1.09,1.44)	1.36 (1.19,1.56)
** **	**Avoids talking about work situation in social contexts**	**n(%)**	270 (67.5%)	54 (55.1%)	56 (57.7%)	78 (74.2%)	82 (82%)
		**PR (CI95%)**	–	ref	1.04 (0.86,1.25)	1.17 (0.99,1.38)	1.30 (1.12,1.51)
		**aPR (CI95%)**	–	ref	1.03 (0.81,1.31)	1.25 (1.02, 1.55)	1.34 (1.09,1.65)
	Financial constraints*						
** **	**Difficulties in managing regular expenses**	**n (%)**	151 (37.7%)	28 (27.7%)	27 (27.3%)	44 (43.6%)	52 (52%)
		**PR (CI95%)**	–	ref	0.98 (0.63,1.54)	1.57 (1.07,2.31)	1.87 (1.30,2.71)
		**aPR (CI95%)**	–	ref	1.07 (0.68,1.68)	1.72 (1.18,2.50)	2.26 (1.55,3.30)
** **	**Inability to afford social activities**	**n(%)**	334 (83.5%)	70 (69.3%)	83 (83.8%)	92 (91.1%)	89 (89.9%)
		**PR (CI95%)**	–	ref	1.21 (1.03,1.41)	1.31(1.14,1.52)	1.30 (1.12,1.50)
		**aPR (CI95%)**		ref	1.21 (1.04,1.42)	1.31(1.13,1.51)	1.27 (1.09,1.48)

Abbreviations: PR, prevalence ratio; aPR, adjusted prevalence ratio; CI95, 95% confidence interval; ref, reference value 1.

## Discussion

We describe positive associations between levels of PE and social precarity related to various aspects of working life and living conditions that remained after adjusting for sociodemographic factors.

In this study, the proportion of workers declaring an experience of social precarity related to working life was high on average. Furthermore, the reporting of experiences of social precarity related to working life was higher among the most precarious employees. These findings are in accordance with the current discourse on PE (measured through temporary contracts): Job dissatisfaction and job insecurity are higher among temporary workers than permanent workers in Australia and Europe.^25,26^ Importantly, experiencing work–life precarity and restraints in social activities are hypothesized to be mechanisms by which PE affects health and quality of life.^6^ Therefore, the associations described in our study may have implications for the health and well-being of workers in PE. For example, working under undesired arrangements could produce feelings of guilt and a lack of control, which may impact the well-being of the PE workers.^27^

An unexpected finding was the low prevalence (1 out of 10) of declaration of being locked in a job (the job they are in does not suit them). Intuitively, this finding appears contradictory to the high proportion (7 out of 10), who declared they are locked in an occupation (they prefer to work in something else) and a preference for permanent employment (9 out of 10). However, these opposing results could be due to how workers perceive the Swedish labor market. Workers in PE may think that they have to settle for whatever job is offered: either take a low-quality job or do not have a job at all, because of the limited job opportunities available.^27^ Another explanation could be the way the question was framed; workers may respond based on different aspects of their job situation (employment arrangements, working hours, tasks, and workmates) rather than their overall experience.

We report a remarkably high prevalence of financial constraints and restraint in social activities among workers in PE, with higher rates with increasing PE. This lack of engagement in social activities may lead to social isolation.^8,28^ Two previous cross-sectional studies conducted in Canada concluded that workers in PE (measured through a multidimensional index) earned lower wages and experienced social isolation.^28^ Therefore, similar conclusions are obtained for individuals working in Canada and Sweden: PE could create barriers and restrict the social activities of workers. Social isolation affects physical and mental health and has been associated with increased all-cause mortality and with cardiovascular and mental disease.^29,30^ Therefore, based on the social nature of humans, the associations found in this study may as well have implications for the well-being of the workers.

Further, we describe that 3 out of 10 precariously employed workers reported difficulties managing regular expenses. Material deprivation may directly affect the physical and mental health of the PE workers, such as poorer self-perceived health,^31^ stress, sleep disturbances, and depression-related symptoms.^27^ Also, it may impact other social determinants of health, such as access to health care, adverse lifestyles, and unhealthy housing conditions.^7^

In our study, the majority of precarious employees were young, born outside of Sweden, and had a lower disposable family income. This finding is in accordance with European data highlighting that young and immigrant workers are most affected by PE.^5,32,33^

Our results suggest that higher levels of PE are slightly more prevalent among men. Sex differences have been suggested in the PE discourse. For example, an empirically based study using data from the 2005 European Working Conditions Survey^32^ found that male and female workers experienced different forms of PE: Women were more prevalent in “precarious unsustainable” jobs (low employment quality characterized for involuntary part-time employment), whereas men were more prevalent in the “precarious intensive” jobs (low employment quality characterized for long working hours). However, due to this study's theoretically based approach for measuring multidimensional PE, we did not explore typologies of PE, which could explain the differences in the distributions among male and female workers. Further, a high proportion of workers included in our study were young. Previous studies have observed few differences between men and women at younger ages;^5^ so, this may also explain the proportion of PE according to sex described in our article.

Our findings of how PE is distributed across different sociodemographic characteristics (not gender) are consistent with previous literature, which suggests that the use of EPRES-Se for measuring PE in the Swedish population is consistent.

Furthermore, workers with higher levels of precariousness reported having had multiple jobs in the past and difficulties piecing several jobs together. Holding multiple jobs has been considered part of the PE definition by the International Labour Organization (ILO)^34^ and was found to be associated with occupational injuries in a systematic review.^35^ Multiple job holding is frequently understood as indicating insufficient income or employment instability and thus, although being debated,^36,37^ our results could support the notion that multiple job holding should be considered when defining multidimensional PE.

This study has some limitations. First, we cannot rule out reverse causation for some of the outcomes, specifically for “having difficulties in managing regular expenses for the past 12 months.” For example, it is possible that some individuals had financial difficulties leading them into PE, or it could be the other way around: Because they were precarious employees, they then suffered from financial strain. Next, we cannot disregard the common method bias (self-reported exposure and outcomes measured at one time point). Despite this, our results agree with previous studies. Longitudinal studies are necessary to validate our findings and to understand the mechanisms and effects of multidimensional PE on social precarity and in various contexts.

In addition, the included individuals may not be representative of the population of nonstandard workers in the Stockholm county. The PREMIS study, however, aimed to recruit individuals that are difficult to reach and not well-captured in other surveys, even at the expense of lower external validity. We also conducted analyses using the RDS weights (which account for oversampling of individuals included in large social networks) and no critical differences between the unweighted and weighted results were observed (data not shown).

Despite the temporariness dimension of EPRES-Se needing revision,^21^ a strength of the study is the use of a tool for measuring PE through a multidimensional approach. EPRES has been previously used in Spanish,^38^ Catalan,^39^ Chilean,^40^ and a Swedish working population.^21^

## Conclusions

To our knowledge, this is the first study in a Nordic welfare state exploring the social precarity associated with PE. Our findings suggest that workers in PE experience social precarity, stressing that PE may have negative effects on well-being. Further studies should analyze these findings in other contexts and explore the mediating effect of social precarity in the relation between PE, health, and well-being of workers.

## Supplemental Material

sj-docx-1-joh-10.1177_00207314211051880 - Supplemental material for The Relation Between Precarious Employment Arrangements and Social Precarity: Findings from the PREMIS Study in Stockholm, Sweden Click here for additional data file. Supplemental material, sj-docx-1-joh-10.1177_00207314211051880 for The Relation Between Precarious Employment Arrangements and Social Precarity: Findings from the PREMIS Study in Stockholm, Sweden by Nuria Matilla-Santander, Johanna Jonsson, Bertina Kreshpaj, Cecilia Orellana, Joan Benach, Kathryn Badarin, Bo Burström, Alejandra Vives, Katarina Kjellberg, Susanne Strömdahl, Gun Johansson, Per-Olof Östergren and Theo Bodin in International Journal of Health Services
