# Supplementary material for: The Relation Between Precarious Employment Arrangements and Social Precarity: Findings from the PREMIS Study in Stockholm, Sweden
Source: Int J Health Serv. 2021 Nov 24;52(2):201–11. doi: 10.1177/00207314211051880 (PMC8894623; doi:10.1177/00207314211051880)
Supplement: sj-docx-1-joh-10.1177_00207314211051880 - Supplemental material for The Relation Between Precarious Employment Arrangements and Social Precarity: Findings from the PREMIS Study in Stockholm, Sweden [file sj-docx-1-joh-10.1177_00207314211051880.docx]

**Supplementary Material**

Figure S1. Directed Acyclic Graph (DAG) of the association between precarious employment arrangements and social precarity. (Page 2)

Table S1. Associations of social precarity per unit increase of EPRES score. (Page 3)

Figure S1. Directed Acyclic Graph (DAG) of the association between precarious employment arrangements, social precarity, and related variables.


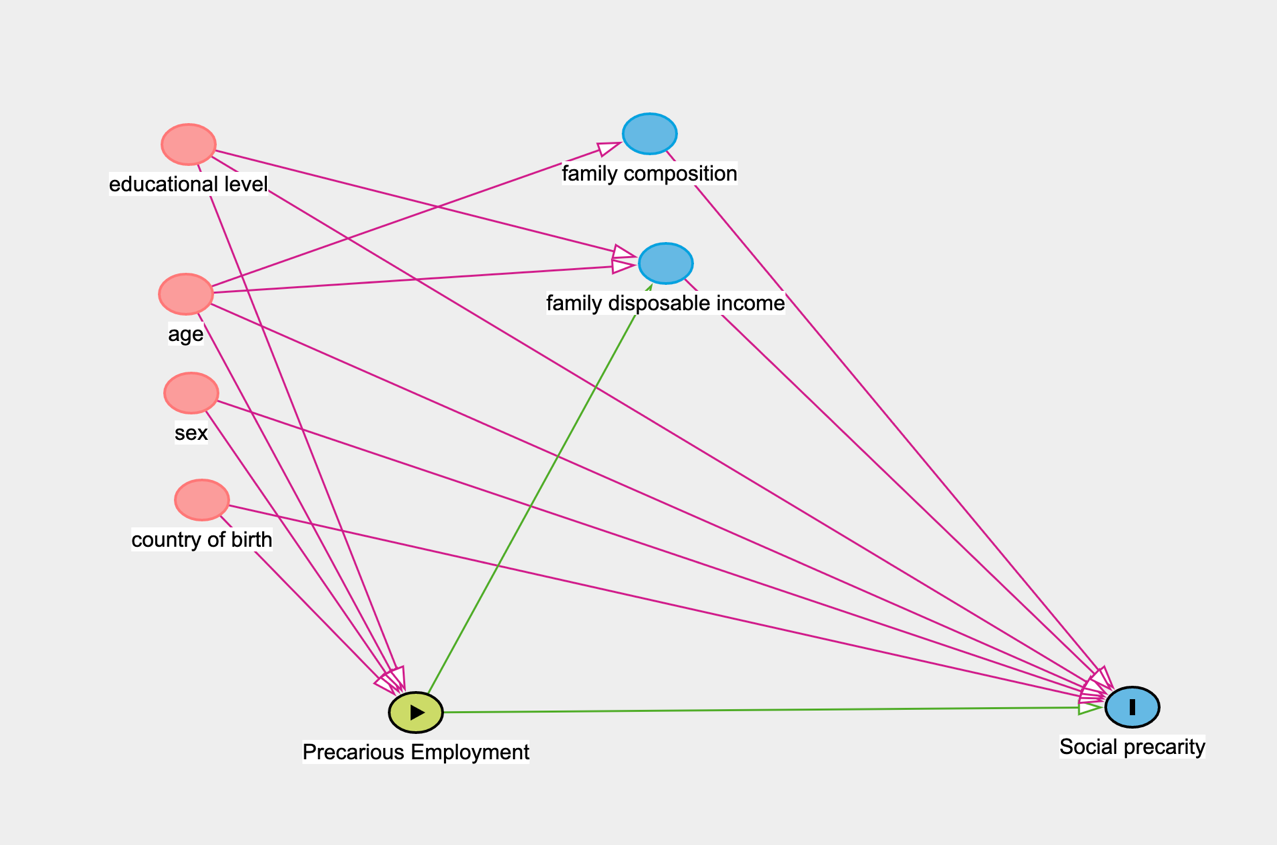


Note: Precarious employment (PE) may cause social precarity through the inability to participate in social activities, financial strain, work–life precarity, and other mediator variables not specified in the path. Further, sociodemographic variables (educational level, sex, age, etc.) directly affect the odds of being precariously employed and suffering social precarity. According to the drawn assumptions in the DAG, the minimal sufficient adjustment sets for estimating the total effect of PE on social precarity are age, sex, county of birth, and level of education.

Table S1. Associations of social precarity per one-unit increase of EPRES-Se score

|  | |  |  |
| --- | --- | --- | --- |
|  |  | **EPRES-Se score** | |
|  |  | **PR (CI 95%)** | **aPR (CI 95%)*** |
| Social precarity related to working life | **Being locked in a job** | 3.30 (2.05–5.32) | 4.35 (2.71–6.99) |
|  | **Would rather have permanent employment** | 1.07 (1.02–1.13) | 1.09 (1.03–1.15) |
|  | **Being locked in an occupation** | 1.27 (1.12–1.44) | 1.20 (1.05–1.36) |
|  | **Difficult to piece several jobs together** | 1.24 (1.12–1.38) | 1.27 (1.14–1.43) |
| Social precarity related to living conditions | **Restraint in social activities** |  |  |
|  | **Cannot participate in social activities because of work** | 1.25 (1.14–1.36) | 1.24 (1.13–1.37) |
|  | **Avoids talking about work situation in social contexts** | 1.41 (1.23–1.61) | 1.38 (1.19–1.60) |
|  | **Financial constraints** | **EPRES-Se score**  **(not including the wages dimension)** | |
|  | **Difficulties in managing regular expenses** | 1.81 (1.39–2.37) | 2.02 (1.55–2.63) |
|  | **Not being able to afford social activities** | 1.18 (1.08–1.29) | 1.22 (1.10–1.36) |

Note: *aPR (adjusted Prevalence Ratios) for sex, age (continuous), level of education, and country of origin.

EPRES-Se: Swedish Employment Precariousness Scale
